# CT: the imaging of choice in the diagnosis of coronary artery fistulae

**DOI:** 10.1259/bjrcr.20150492

**Published:** 2016-07-28

**Authors:** Anam Ali, Jonathan Colledge, Iyengar Sri, Constantinos Missouris

**Affiliations:** ^1^Department of Radiology, Frimley Health NHS Foundation Trust, Slough, UK; ^2^Department of Radiology, Barts Health NHS Foundation Trust, London, UK; ^3^International Centre for Circulatory Health, Imperial College, London, UK

## Abstract

Coronary artery fistulae are rare abnormal communications between coronary arteries and cardiac chambers or great vessels. We report a patient with a complex congenital fistula between the pulmonary artery and both the left anterior descending and the right coronary artery, originally diagnosed on routine coronary angiography and subsequently followed up and further elucidated with CT coronary angiography.

## Case report

A 75-year-old female with well-controlled hypertension presented with a 2-week history of palpitations. On examination, her pulse was regular at 76 bpm, blood pressure was 150/70 mmHg and auscultation revealed a continuous murmur heard over the precordium. She had no signs of heart failure. Resting electrocardiogram (ECG) was within normal limits, and the chest X-ray showed prominent pulmonary arteries on the left side, but normal heart size and clear lung fields. Transthoracic echocardiogram revealed normal left ventricular systolic function with normal ejection fraction at 65%. There was impaired relaxation with Grade 1 diastolic dysfunction. The right ventricular structure and function were normal and there were no significant valvular lesions, with only trivial mitral and tricuspid regurgitation. 24-h Holter monitoring demonstrated a normal heart rate profile, with frequent isolated ventricular and supraventricular ectopic beats, and one episode of broad complex tachycardia of 4 beats. She had blunted heart rate response to exercise and the maximum heart rate was 102 bpm despite completing Stage 3 of the normal Bruce protocol. At peak exercise, the blood pressure dropped from 150/94 mmHg to 90/68 mmHg, and this was associated with presyncope.

**Figure 1.**
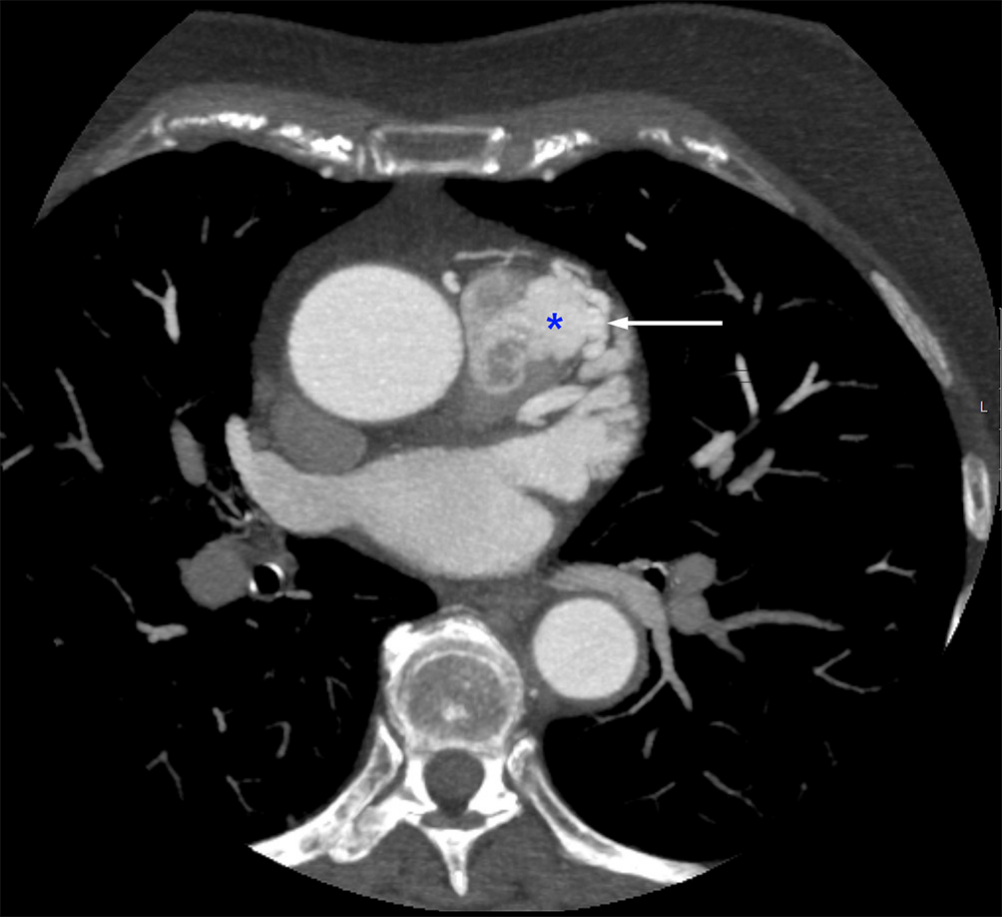
Axial image demonstrating the coronary artery fistula segment communicating with the pulmonary trunk (white arrow) and the blush of iodinated contrast into the pulmonary artery (blue asterisk).

**Figure 2.**
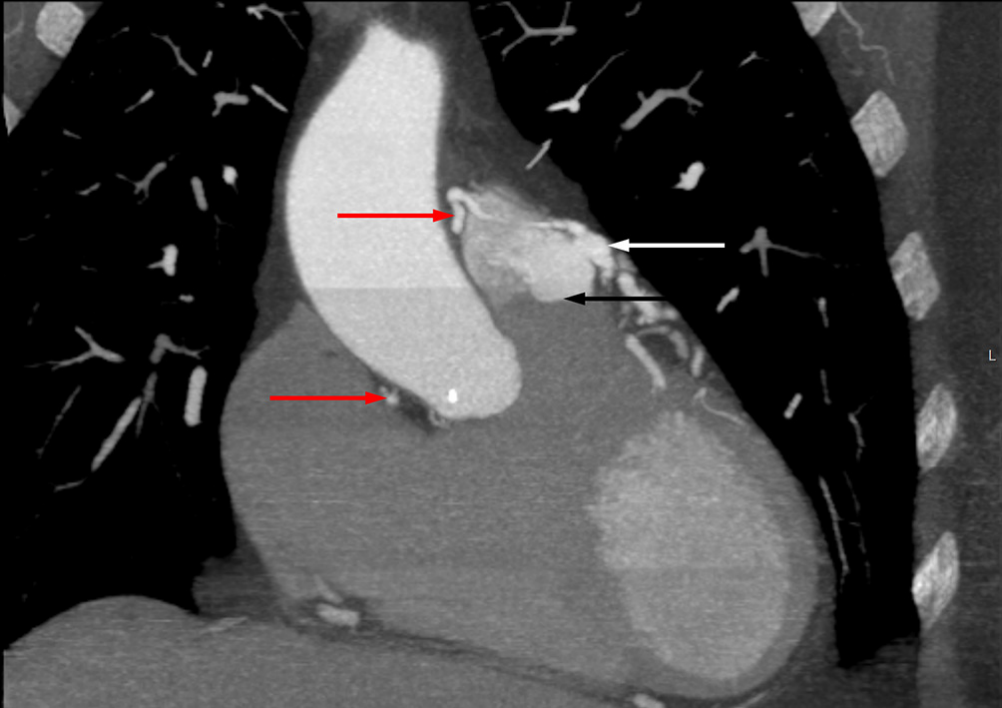
Coronal image demonstrating the communication between the fistula and the pulmonary trunk (white arrow) just above the pulmonary valve (black arrow) and the communicating vessel (red arrows) extending to the right coronary artery.

**Figure 3.**
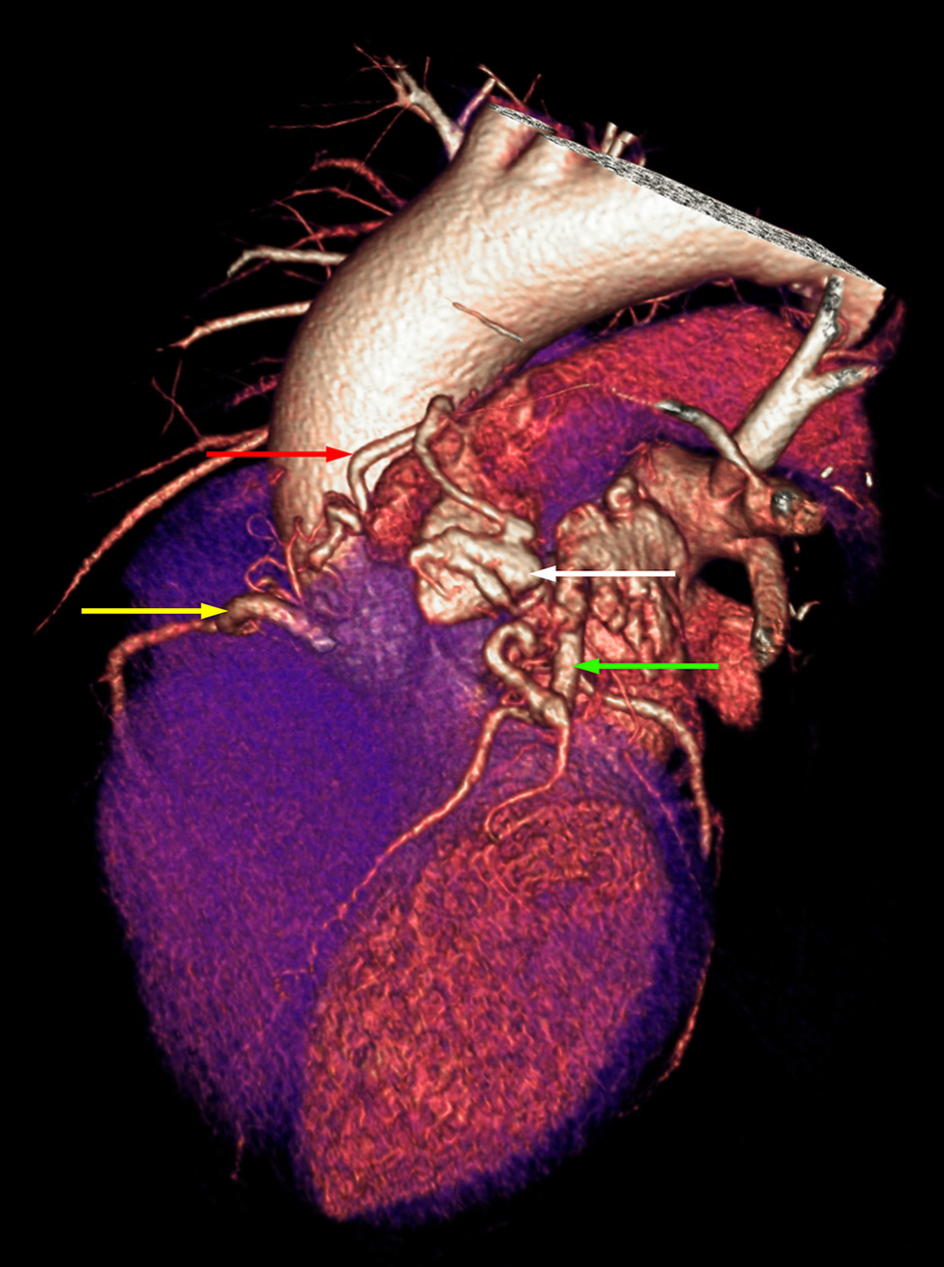
Volume-rendered reconstruction image demonstrating the coronary artery fistula segment communicating between the pulmonary trunk (white arrow) and the left anterior descending artery (green arrow) and the communicating vessel (red arrow) extending to the right coronary artery (yellow arrow).

Coronary angiography showed a large communication between the left anterior descending (LAD) artery and the main pulmonary artery. In addition, there was another smaller communication that appeared to run from the right coronary artery (RCA) to the pulmonary artery. She was subsequently referred for CT coronary angiography (CTCA), which was performed using a 64-slice multidetector CT scanner using prospective ECG-gated acquisition. This confirmed the presence of a large complex fistula, between the main pulmonary conus and both the LAD and proximal RCA, with cavernous malformation of the fistula segment to the LAD (Figures 1–3). There were two branches of the fistula, the larger branch communicating to the LAD and a further smaller branch to the RCA. The scan also showed minimal plaque burden in the RCA. The patient was reluctant to consider surgical correction of the fistulae, and as she was largely asymptomatic, medical management was pursued. 13 years following the initial diagnosis, she remains asymptomatic with normal left and right ventricular echocardiographic parameters on follow-up.

## Discussion

Coronary artery fistulae (CAF) are rare, found in 0.1–0.2% of patients undergoing coronary angiography and comprise 13% of coronary anomalies.^1–5^ They were first described in 1865^6^ and represent abnormal communications between coronary arteries and cardiac chambers (coronary–cameral fistulae) or great vessels.^7^ The majority are congenital; however, acquired fistulae have been reported following trauma, myocardial infarction, coronary artery bypass grafting and percutaneous coronary intervention. Spontaneous CAF are rare.^8^ The fistulae typically arise from the RCA (50–60%), less often from the left coronary artery (25–42%), and in 5% of cases, from both coronary arteries. Approximately 90% drain into right-sided structures of the heart. Drainage into the left-sided structures is less common.^7,9^

The majority of CAF are asymptomatic, follow a benign course and rarely compromise myocardial blood flow. They can later present with an atypical cardiac murmur, fatigue, dyspnoea or angina. Complications include pulmonary hypertension owing to left-to-right shunt, myocardial infarction secondary to coronary steal, congestive cardiac failure, endocarditis, aneurysm formation, thrombosis, arrhythmias and spontaneous rupture.^7–9^

Most asymptomatic patients are medically managed with close follow-up. Intervention using the surgical or transcatheter route is recommended in patients with large fistulae regardless of symptomatology or with small-to-moderate fistulae presenting with myocardial ischaemia, arrhythmia, unexplained ventricular systolic or diastolic dysfunction, or enlargement or endarteritis.^10^

Coronary angiography has traditionally been used to diagnose CAF; however, this invasive procedure is not optimal as it carries inherent risks, including a 0.1% risk of mortality and vascular complications.^11^ Furthermore, the precise course of CAF anatomy can be difficult to delineate, particularly the drainage sites, which can be diluted by the contrast medium, and the images may be ambiguous when faced with anomalous, abnormal and tortuous blood vessels.^9,11–12^

CTCA using prospective or retrospective ECG-gated acquisitions provides an alternative approach, and is now widely being used to image coronary arteries and their anomalies with greater accuracy and reliability.^9,11–14^ This non-invasive imaging technique has led to increasing numbers of CAFs being found incidentally. CTCA can reconstruct optimal three-dimensional images and accurately evaluate structure, including the presence of aneurysmal dilatation and thrombus formation using similar or lower contrast volumes than conventional coronary angiography.^15^ Moreover, the high spatial and contrast resolution images provide an excellent overview of the cardiac and vascular anatomy, which is useful in helping surgeons understand anatomical intricacies prior to surgery.^9,11^ In addition, temporal resolution can be optimized to image at high heart rates, using a variety of hardware and reconstruction techniques, including high-pitch scanning, large detector banks, half-scan reconstruction and multicycle reconstruction.

Our case demonstrates the role of CTCA as an important imaging modality in clearly delineating the anatomy, true course and extent of CAF. CTCA should be considered as the investigation of choice in the diagnosis and follow-up of patients suspected of having CAF.

## Learning points

1. CTCA is a non-invasive imaging modality that is being used to delineate CAF anatomy with greater accuracy and reliability.
2. Optimal CTCA images can be obtained using similar or lower contrast volumes than conventional coronary angiography, but with the advantage of clear delineation of the full course of the fistula and the related structures (in this case, the main pulmonary artery).
3. CTCA should be considered as the investigation of choice in the diagnosis and follow-up of patients suspected of having a CAF.

## Consent

Written informed consent for the case to be published (including images, case history and data) was obtained from the patient for publication of this case report.
